# Supramolecular Polymorphism of (G_4_C_2_)_n_ Repeats Associated with ALS and FTD

**DOI:** 10.3390/ijms22094532

**Published:** 2021-04-26

**Authors:** Melani Potrč, Nerea Sebastián, Miha Škarabot, Irena Drevenšek-Olenik, Lea Spindler

**Affiliations:** 1 Faculty of Natural Sciences and Mathematics, University of Maribor, Koroška 160, 2000 Maribor, Slovenia; melani.potrc@student.um.si; 2 Department of Complex Matter, Jožef Stefan Institute, Jamova 39, 1000 Ljubljana, Slovenia; nerea.sebastian@ijs.si (N.S.); irena.drevensek@ijs.si (I.D.-O.); 3 Department of Condensed Matter Physics, Jožef Stefan Institute, Jamova 39, 1000 Ljubljana, Slovenia; miha.skarabot@ijs.si; 4 Faculty of Mathematics and Physics, University of Ljubljana, Jadranska 19, 1000 Ljubljana, Slovenia; 5 Faculty of Mechanical Engineering, University of Maribor, Smetanova 17, 2000 Maribor, Slovenia

**Keywords:** DNA-quadruplex, G-wires, d(GGGGCC) repeats, self-assembly, dynamic light scattering, AFM

## Abstract

Guanine-rich DNA sequences self-assemble into highly stable fourfold structures known as DNA-quadruplexes (or G-quadruplexes). G-quadruplexes have furthermore the tendency to associate into one-dimensional supramolecular aggregates termed G-wires. We studied the formation of G-wires in solutions of the sequences d(G_4_C_2_)_n_ with *n* = 1, 2, and 4. The d(G_4_C_2_)_n_ repeats, which are associated with some fatal neurological disorders, especially amyotrophic lateral sclerosis (ALS) and frontotemporal dementia (FTD), represent a challenging research topic due to their extensive structural polymorphism. We used dynamic light scattering (DLS) to measure translational diffusion coefficients and consequently resolve the length of the larger aggregates formed in solution. We found that all three sequences assemble into longer structures than previously reported. The d(G_4_C_2_) formed extremely long G-wires with lengths beyond 80 nm. The d(G_4_C_2_)_2_ formed a relatively short stacked dimeric quadruplex, while d(G_4_C_2_)_4_ formed multimers corresponding to seven stacked intramolecular quadruplexes. Profound differences between the multimerization properties of the investigated sequences were also confirmed by the AFM imaging of surface films. We propose that π-π stacking of the basic G-quadruplex units plays a vital role in the multimerization mechanism, which might be relevant for transformation from the regular medium-length to disease-related long d(G_4_C_2_)_n_ repeats.

## 1. Introduction

Although DNA sequences forming guanosine quadruplexes (G-quadruplexes) have been extensively studied for several decades, new folding patterns in widely investigated strands are still being discovered [1,2]. Therefore, the conformational polymorphism of G-quadruplex structures remains an important research challenge. Quite frequently, in standard conditions, several quadruplex folds simultaneously coexist in a solution, which makes a precise determination of different folding topologies very difficult. Such behavior is also typical for GGGGCC (G_4_C_2_) repeat sequences associated with neurological disorders amyotrophic lateral sclerosis (ALS) and frontotemporal dementia (FTD) [3,4,5,6]. The most common mutation related to these diseases is an increased number of d(G_4_C_2_) repeats within the *C9orf72* gene. Individuals with ALS and FTD generally have hundreds to thousands of G_4_C_2_ repeat units, while healthy individuals typically have less than 25 of them [7,8]. The molecular mechanisms through which these repeat expansions and their quadruplex structures cause the neurodegenerative modifications are not yet known. Nevertheless, some potential therapeutics targeting them are already being investigated [9,10].

**Figure 1:**
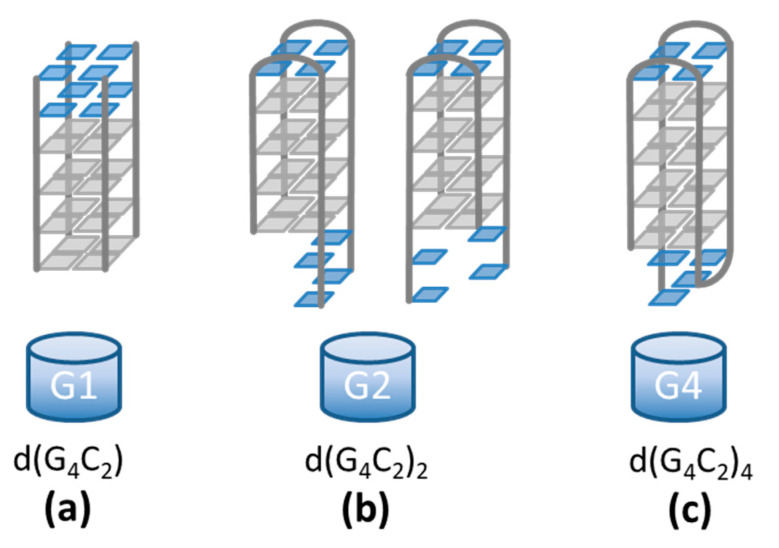
d(G_4_C_2_)_n_ sequences form quadruplex structures in which four guanines (gray squares) associate via Hoogsteen-type hydrogen bonding. (**a**) The d(G_4_C_2_) sequence forms a tetrameric symmetric quadruplex with 3′-CC overhangs (blue squares) [4]. (**b**) The d(G_4_C_2_)_2_ sequence shows a high polymorphism with the exact structures still being unknown [5]. Two possible dimeric folds are depicted. (**c**) The d(G_4_C_2_)_4_ sequence is believed to form a unimolecular antiparallel quadruplex with edgewise loops [11,12,13].

A recent study indicated that, in addition to their conformational diversity, G-quadruplex structures from d(G_4_C_2_) repeats are prone to supramolecular assembly beyond the simple G-quadruplex formation [4]. This feature brings additional difficulties to the experimental determination of the exact G-quadruplex folding topologies. To tackle this problem, different approaches were developed, like 8Br-dG substitution [11] or anion exchange chromatography [5]. Only a few of the possible G-quadruplex folds could be pinned down. For d(G_4_C_2_), the basic building block is a parallel tetrameric G-quadruplex (Figure 1a) [4]. For d(G_4_C_2_)_2,_ several folding topologies are possible, but most of them are dimeric quadruplexes (Figure 1b) [5]. For d(G_4_C_2_)_4,_ a monomolecular antiparallel quadruplex was identified as the predominant species (Figure 1c) [11,12]. A possible role of d(G_4_C_2_) supramolecular assembly, in particular multimerization of the associated G-quadruplexes, in the development of ALS and FTD is, however, still a fully open problem.

This work is focused on the investigation of supramolecular structures formed in solutions of d(G_4_C_2_), d(G_4_C_2_)_2,_ and d(G_4_C_2_)_4_. For this purpose, we used two methods that are not routinely used in DNA-quadruplex research, namely dynamic light scattering (DLS) and atomic force microscopy (AFM). Both of them give information on the size and shape of the objects present in the system, while they cannot resolve atomistic details of their internal structure. With DLS measurements, we determined the length of the larger d(G_4_C_2_)_n_ aggregates in the solution, while with AFM, we tested the presence of such macromolecular assemblies in the drop cast films deposited from the solution onto a solid substrate. The obtained results revealed a surprising ability of the shortest of the three investigated sequences, i.e., d(G_4_C_2_), to form remarkably long G-quadruplex-based assemblies associated with the so-called guanine wires (G-wires) [14]. In contrast, the other two sequences, namely d(G_4_C_2_)_2_ and d(G_4_C_2_)_4_, formed much shorter assemblies.

## 2. Results

### 2.1. Dynamic Light Scattering

**Figure 2:**
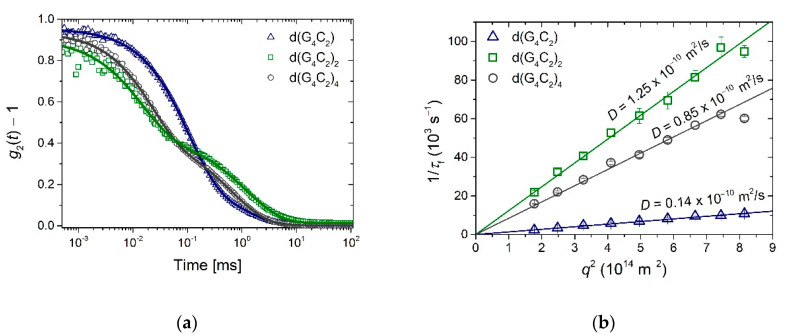
DLS measurements: (**a**) Autocorrelation curves taken at *ϑ* = 90° clearly reveal very slow dynamics of the fast mode in solution of the d(G_4_C_2_) sequence. (**b**) The measured dependencies of the inverse relaxation time of the fast mode 1/*τ*_f_ versus *q*^2^ were used to calculate the translational diffusion coefficients *D* (Equation (2)). Quite notably, there is no evident correlation between the obtained values of *D* and the number of G_4_C_2_ repeats in the investigated sequence, which indicates that these sequences exhibit very diverse self-assembly patterns.

Although the three investigated sequences seem to be very similar, DLS measurements revealed their profoundly different self-assembly processes. Before the measurements, freshly prepared solutions were left to equilibrate for a week to make sure that they reached thermodynamically stable structures. After measuring the autocorrelation functions *g*_2_(*t*) (Figure 2a) at several scattering angles and fitting them to Equation (1) (see Materials and Methods section), the obtained inverse relaxation times of the fast mode 1/*τ*_f_ were plotted versus *q*^2^ (Figure 2b). The slope of the corresponding linear fits was used to deduce the diffusion coefficients in accordance with the Equation (2). Surprisingly, the shortest sequence, namely d(G_4_C_2_), exhibited the lowest value of *D* = (0.14 ± 0.02) ∙ 10^−10^ m^2^/s, indicating extremely long rod-like assemblies. The sequence d(G_4_C_2_)_2_ exhibited the highest value of *D* = (1.25 ± 0.02) ∙ 10^−10^ m^2^/s, while the sequence d(G_4_C_2_)_4_ exhibited an intermediate value of *D* = (0.85 ± 0.02) ∙ 10^−10^ m^2^/s. The measurements were also repeated a week later, and, within the experimental error, the same values of the diffusion coefficients were obtained. To estimate the length distribution of aggregates an average stretch factor *s* for all scattering angles was calculated. For completely monodisperse scatterers the expected value is *s* = 1, while lower *s* values are typical for more polydisperse objects. We obtained *s* = 0.86 ± 0.01 for d(G_4_C_2_), *s* = 0.98 ± 0.02 for d(G_4_C_2_)_2_ and *s* = 0.90 ± 0.02 for d(G_4_C_2_)_4_ indicating quite narrow size distributions for all three sequences.

**Figure 3:**
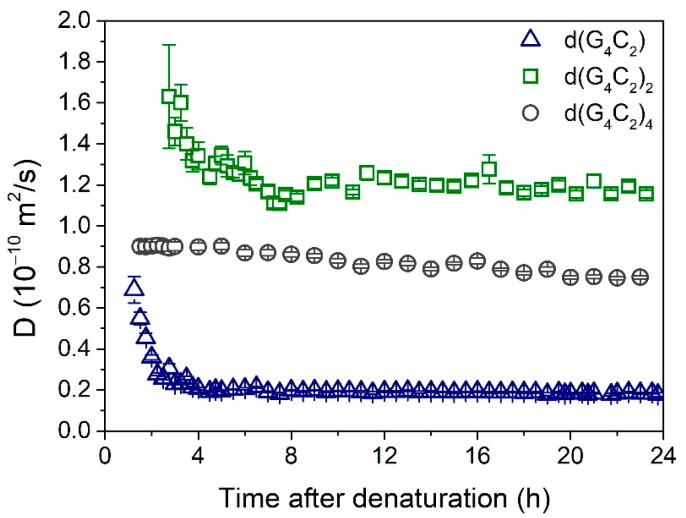
Time dependences of the diffusion coefficient after thermal denaturation (performed at *t* = 0 h). The highest values of *D* observed in solution of d(G_4_C_2_)_2_ indicate the presence of small structures, most probably two stacked bimolecular quadruplexes. The intermediate values of *D* observed in solution of d(G_4_C_2_)_4_ are probably associated with higher-order quadruplexes. The lowest values of *D* observed in solution of d(G_4_C_2_) signify a formation of extremely long G-wires created by stacking of individual parallel quadruplex units.

Additional information on the assembly process was obtained by denaturation of the solutions and subsequent monitoring of the DLS signal. Before DLS measurements, the denatured samples were left for one hour to cool down to the ambient temperature. The results are shown in Figure 3. In solution of d(G_4_C_2_), long assemblies started to form already during the cooling process, and their growth continued for another 4 h, with the final value of *D* getting below 0.2 ∙ 10^−10^ m^2^/s. In contrast, in solution of d(G_4_C_2_)_4_, the observed value of *D* ≈ 0.9 ∙ 10^−10^ m^2^/s was practically constant during the first 6 h. Afterward, it very slowly decreased with the increasing time, gradually reaching the final value of *D* ≈ 0.8 ∙ 10^−10^ m^2^/s. In solution of d(G_4_C_2_)_2_, the self-assembly process was rather slow, and it took more than 2 h before enough scattering objects were formed to produce a reasonable DLS signal. The initially detected very large value of *D* ≈ 1.6 ∙ 10^−10^ m^2^/s indicates that at first very small folds were formed, which in the next 6 h developed into the final structures with *D* ≈ 1.2 ∙ 10^−10^ m^2^/s.

### 2.2. Atomic Force Microscopy

**Figure 4:**
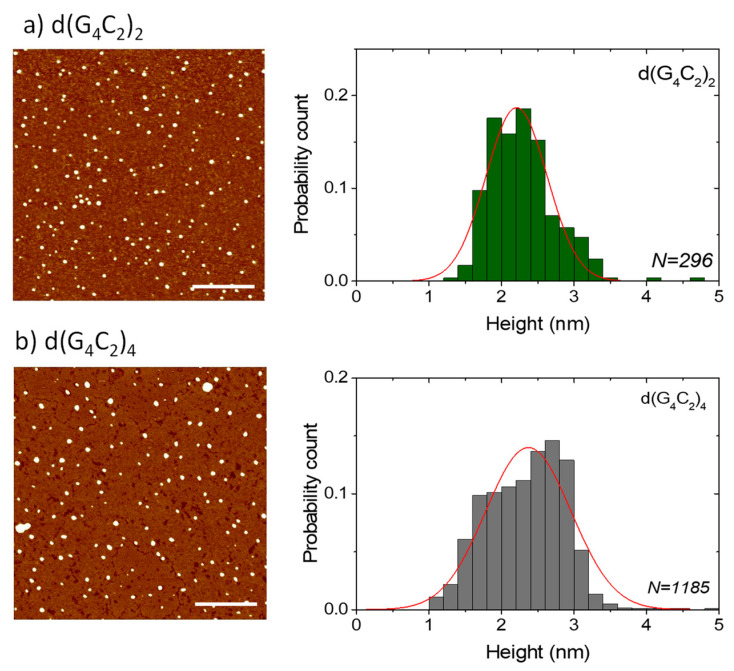
AFM images of small globular islands formed in drop-cast surface films of (**a**) d(G_4_C_2_)_2_ and (**b**) d(G_4_C_2_)_4_. The height distribution of the structures is given in the right panels and shows an average height of around 2.4 nm. The lines are guides to the eye. The scale bars in the AFM images correspond to 500 nm.

AFM was used to additionally test the extent of the self-assembly of the three investigated sequences and especially to confirm the ability of the shortest hexanucleotide d(G_4_C_2_) to form extremely long G-wires. AFM images of the films formed from the solutions of d(G_4_C_2_)_2_ and d(G_4_C_2_)_4_ reveal numerous small globular islands with variable sizes (Figure 4). All islands have a very similar height of *h* = (2.4 ± 0.4) nm for d(G_4_C_2_)_2_ and *h* = (2.3 ± 0.6) nm for d(G_4_C_2_)_4_. Although this height is smaller than the actual G-quadruplex diameter in solution and also smaller than the G-wire height detected in solution AFM imaging [15], it is the usual value found in AFM imaging of air-dried G-quadruplexes on mica substrates [16,17]. While in AFM imaging, the height of the structures can be measured with a ± 0.1 nm precision, the lateral spatial resolution is much worse due to the tip curvature. Similar surface structures, however, were observed in the films formed by parallel tetrameric G-quadruplexes from the *Tetrahymena* telomeric repeat sequence d(TG_4_T) [18] and the G-rich oligonucleotides d(TG_8_T) and d(TG_9_) [19].

**Figure 5:**
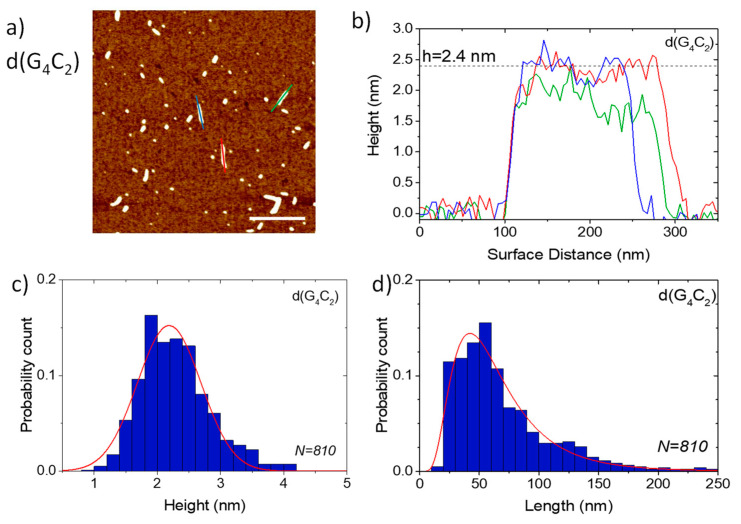
(**a**) AFM image of a drop cast film of d(G_4_C_2_). The scale bar corresponds to 500 nm. (**b**) The height profiles along three single G-wires (see colored lines in (**a**)). (**c**) The length and (**d**) the height distribution of G-wires. The lines are guides to the eye. Length analysis was limited to objects above 1 nm and elongated in shape.

In addition to spherical islands, surface structures from d(G_4_C_2_) also revealed the formation of long wire-like assemblies with lengths spanning from few nanometers up to around one hundred nanometers (Figure 5). The actual G-wire lengths were around 15 nm smaller than seen by AFM imaging due to the finite tip size effect [20]. Despite large differences in their lengths, all wires showed the same relatively homogeneous height of *h* = (2.3 ± 0.6) nm. This height is typical for G-wires lying flat on the mica surface and being buttressed by magnesium ions [21,22,23]. The fact that d(G_4_C_2_)_2_ and d(G_4_C_2_)_4_ did not form such G-wires, is in agreement with their limited stacking ability in solution as witnessed by the DLS measurements.

## 3. Discussion

Combining the obtained DLS and AFM results with the previously published work on d(G_4_C_2_)_n_ repeats [4,5,11,12,24,25,26], we propose the following models for their self-assembly.

### 3.1. Self-Assembly of d(G_4_C_2_)

**Figure 6:**
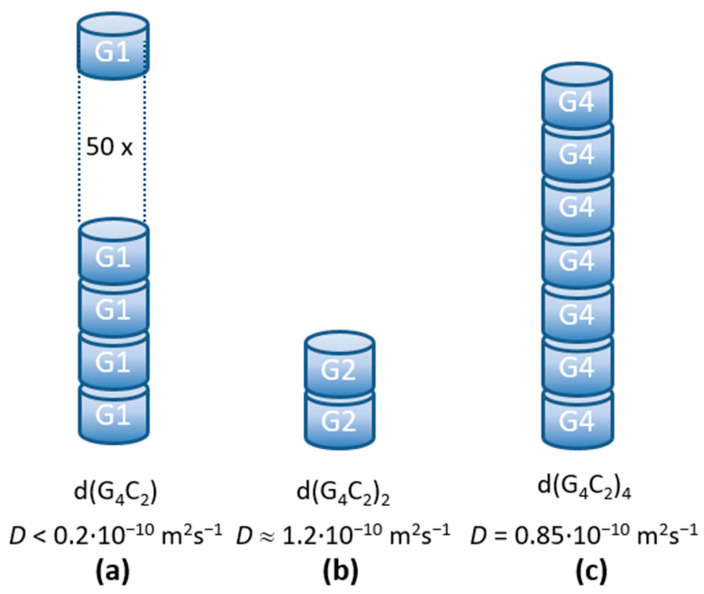
The model for the largest diffusing structures in the solutions [38] as deduced from the DLS data. The quadruplexes from d(G_4_C_2_), d(G_4_C_2_)_2_, and d(G_4_C_2_)_4_ show very different stacking ability with d(G_4_C_2_) forming the longest (**a**), d(G_4_C_2_)_2_ the shortest (**b**) and d(G_4_C_2_)_4_ intermediate sized G-wires (**c**). All aggregates determined by DLS are longer than previously reported with other methods.

For the d(G_4_C_2_) hexanucleotide the basic structural unit was proposed to be a tetra-molecular parallel G-quadruplex [4] (Figure 1a). The NMR spectra for this sequence already indicated formation of larger assemblies, but with NMR it was not possible to comprehend the extent of the stacking process. As it turns out, the d(G_4_C_2_) hexanucleotide forms exceptionally long G-wires, that have never been reported for this sequence before. The diffusion coefficient *D* = (0.14 ± 0.02) ∙ 10^−10^ m^2^/s is outside the limits of the Tirado and Garcia de la Torre theory (*L*/*d* > 30). The length of the aggregates in solution is believed to be larger than ~80 nm, which corresponds to more than ~50 stacked basic G-quadruplex units (Figure 6a). Such results are supported by the AFM findings showing long wire-like structures up to one hundred nanometers. Although stacking of G-quadruplexes was reported for a number of G-rich DNA sequences [27,28,29,30], such extremely long aggregates have been until now observed only for 5′-dGMP in the form of diammonium salt [31,32], for the *Tetrahymena thermophila* telomeric sequence d(G_4_T_2_)_4_, and Human telomeric sequence d(TTAG_3_)_4_ in the presence of Sr^2+^ ions [33,34]. From our measurements, the exact mechanism of the stacking cannot be resolved, but there exist a few possible scenarios. Šket et al. [4] proposed stacking of two basic units through 5′-5′ interfaces. Such dimerization through cofacial stacking was indeed proven to be energetically favorable to 5′-3′ or 3′-3′ stacking [35]. This dimerized structure, however, is flanked by four CC overhangs on each end. In order to continue the stacking process, these CC overhangs need to re-arrange themselves. One possibility is the exclusion of the 3′ flanking overhangs [36]. Another possibility is the formation of a CCCC tetrad that is formed above the last G-tetrad and stabilized by π-π stacking [26]. The stacking of two such loosely formed end surfaces, however, does not seem very probable. An alternative possibility is the energetically less favorable 5′-3′ stacking (head-to-tail) [35] of single G-quadruplexes that could give more stable long aggregates beyond the dimer formation. Whatever the stacking mechanism is, our measurements show that the final G-wires resulting from it are extremely long.

### 3.2. Self-Assembly of d(G_4_C_2_)_2_

DLS data show that d(G_4_C_2_)_2_ repeats have the highest diffusion coefficient of the three studied repeats (Figure 2b) and thus form the shortest aggregates. The first structures detected after denaturation (Figure 3) have a diffusion coefficient of *D* ≈ 1.6 ∙ 10^−10^ m^2^/s. This value is outside the limits of the TGT theory, but, based on previous work [37] it is associated with quadruplex folds having four stacked G-quartets. The structures forming soon after the denaturation are therefore most likely dimeric G-quadruplexes (Figure 1b). Their exact structure is up to date still unknown since G-quadruplexes from d(G_4_C_2_)_2_ exhibit a number of different topologies and are a mix of parallel and antiparallel conformations that are simultaneously existing in the solution. The equilibrium assembly of d(G_4_C_2_)_2_ is reached 6 h after denaturation and has a value of *D* = (1.25 ± 0.02) ∙ 10^−10^ m^2^/s. There are two possible structures that could correspond to such diffusion coefficients: either two stacked dimeric quadruplexes (higher-order structure) or a symmetric tetramer. Both were proven to exist together with the dimeric form in d(G_4_C_2_)_2_ repeat solutions [5]. However, for the parallel tetrameric form it is expected that is should easily form G-wires, which were not observed by the AFM imaging. Thus, the stacked dimeric quadruplex remains the most likely option (Figure 6b) for being the largest aggregate of self-assembled d(G_4_C_2_)_2_ repeats, while the presence of other, smaller, topologies cannot be excluded based on our DLS data.

### 3.3. Self-Assembly of d(G_4_C_2_)_4_

Finally, the aggregates from d(G_4_C_2_)_4_ repeats exhibit translational dynamic features in between the two smaller sequences. Their size is the only one that fits into the TGT theory limits with the measured value of *D* ≈ 0.9 ∙ 10^−10^ m^2^/s. This result is rather surprising, since several papers reported that d(G_4_C_2_)_4_, as well as r(G_4_C_2_)_4_, repeats formed a small unimolecular G-quadruplex [4,11,12,13,24,39]. Only Reddy et al. [24] reported for r(G_4_C_2_)_4_ repeats that they could also form electrophoretically slow migrating structures and confirmed their multimolecular origin. These multimers are most likely stacked monomolecular quadruplexes. Based on the NMR studies, the d(G_4_C_2_)_4_ sequence in K^+^ solutions forms an antiparallel quadruplex with 4 G-quartet planes (Figure 1c). From the TGT theory we can estimate the number of stacked units using Equation (3) with *D* = 0.85 ∙ 10^−10^ m^2^/s (Figure 2b) and consequently obtain the length of the structures to be *L* = 10 nm. With an average stacking height of 0.34 nm this yields 29 G-quartet planes, which corresponds to around seven stacked monomolecular quadruplexes (Figure 6c). Although this finding is a bit surprising, similar higher-order structures were previously reported also for a number of other G-quadruplex forming sequences investigated by DLS measurements and other methods including theoretical modeling [33,34,40,41,42]. We should also note that the diffusion coefficient *D* = 0.85 ∙ 10^−10^ m^2^/s and the corresponding dimensions of the aggregates could be associated with a parallel tetrameric G-quadruplex [34]. All previous work on this sequence, however, indicates that such a tetrameric G-quadruplex in an unlikely fold.

In general, DLS results for d(G_4_C_2_)_2_ and d(G_4_C_2_)_4_ do not rule out the simultaneous existence of smaller, monomolecular, or bimolecular, G-quadruplexes in solution. Unfortunately, AFM imaging for these two sequences did not give more insight into the polymorphism of these smaller structures due to the limitations of AFM imaging in the air [15].

## 4. Materials and Methods

### 4.1. Materials

All three oligonucleotides (d(G_4_C_2_), d(G_4_C_2_)_2,_ and d(G_4_C_2_)_4_) were purchased in lyophilized form from Eurogentec (Seraing, Belgium), purified by HPLC-RP, and desalted on a Sephadex G25 column. The material was dissolved in 100 mM KCl to a final strand concentration of 1 mM, and the aliquots of about 300 µL were put into 5 mm diameter glass capillaries. The denaturation was performed by heating the samples to 95 °C and holding the temperature constant for 5 min. Afterward, the samples were left to slowly cool down to room temperature.

### 4.2. Dynamic Light Scattering

Dynamic light scattering (DLS) measurements were performed using either a He-Ne laser operating at *λ* = 633 nm or a frequency-doubled Nd:YAG laser operating at 532 nm as the light source. For detection of scattered light, a digital correlator (ALV-7002 Multiple Tau) in combination with an avalanche photodiode was used. The scattered light was detected at scattering angles *θ* ranging from 30 to 130°.

The theoretical background and the experimental setup were described in detail in our previous work [43]. In the majority of the measurements, two diffusive dynamic modes were observed that are referred to as the fast and the slow mode. The measured intensity autocorrelation functions were fitted to the following relation [44]:

(1) $$g_{2}\left(t\right)-1={\left(1+j_{d}\left(a_{f}\exp\left(-{\left(\frac{t}{\tau_{f}}\right)}^{s_{f}}\right)+\left(1-a_{f}\right)\exp\left(-{\left(\frac{t}{\tau_{s}}\right)}^{s_{s}}\right)-1\right)\right)}^{2}+y_{0},$$

where *y*_0_ is the baseline correction, *j*_d_ is the ratio between the intensity of the inelastically scattered light and the intensity of total scattered light, *a*_f_ is the amplitude of the fast mode, and *τ*_f_ and *τ*_s_ are the relaxation times of the fast and the slow mode, respectively. The stretch exponent parameters, *s*_f_ and *s*_s_, are associated with the relaxation times distribution and can attain values between 0 and 1.

The translational diffusion coefficient *D* was obtained from the relation:

(2) $$D=\frac{1}{\tau q^{2}},$$

where *q* is the scattering wave vector defined as *q* = (4π*n*/*λ*)sin(*θ*/2), *n* = 1.33 is the solution refractive index, and *λ* the laser wavelength. From the two observed dynamic modes, the fast mode was associated with the diffusion of G-quadruplexes and their multimerized assemblies, while the slow mode was assigned to the diffusion of large, unspecific “clusters”. This mode is generally known as the »slow mode« of polyelectrolyte solutions and was observed in solutions of various polyelectrolytes, including quadruplex-forming G-rich DNA sequences [45,46,47]. However, the associated loose clusters have dimensions in the micrometer range and are believed not to be directly connected to the G-quadruplex formation.

To obtain the size of the diffusing objects from the measured value of *D*, an appropriate theoretical model is needed. For diluted solutions of rod-like particles, the hydrodynamic theory of Tirado and Garcia de la Torre (TGT theory) can be applied [48]. For the length to diameter ratio *p* = *L*/*d* in the range 2 ≤ *p* ≤ 30, the expression for *D* in this theory is given by the interpolation equation

(3) $$D=\frac{k_{B}T}{3\pi \eta L}(\ln p+\nu ),$$

where *ν* is the end-effect correction given as *ν* = 0.312 + 0.565/*p* + 0.100/*p*^2^, *k*_B_ the Boltzmann constant, *T* the solution temperature, and *η* the solvent viscosity. In the experiments, *T* was 296 K, and *η* was taken to be 0.932 mPa⋅s, which is solvent viscosity at this temperature. Previous NMR studies [4] have shown that the basic quadruplex unit of d(G_4_C_2_) does not have any side loops, so the value of *d* = 2.6 nm that takes into account also the hydration sphere around such G-quadruplexes was used [43]. For this case, Equation (3) can be reasonably applied for scattering objects with the lengths in the range 5 nm ≤ *L* ≤ 80 nm corresponding to the diffusion coefficients in the range 1.2 · 10^−10^ m^2^/s ≥ *D* ≥ 0.22 · 10^−10^ m^2^/s.

If a solution contains scattering objects of different sizes, the DLS signal “favors” larger ones since the intensity of scattered light is proportional to the square of the scattering volume [38]. For rod-like particles with the same diameter *d* this gives the ∝ *L*^2^ dependence of the scattered intensity. In case of polymorphic structures in solution, DLS cannot resolve only one of possible coexisting topologies. However, because light scattering cross section increases with the square of the scattering volume, the contribution from larger scatterers predominates in the DLS signal. In addition, one also needs to take into consideration the fact that G-quadruplexes and their assemblies behave as strong polyelectrolytes, and consequently, electrostatic interactions influence their solution dynamics. However, based on previous work [33], we can assume that in 1 mM oligonucleotide solutions with the addition of 100 mM KCl the electrostatic interactions are strongly screened. Therefore, the values of *L* calculated by the use of the Equation (3) are very close to the actual length of the scattering objects.

### 4.3. Atomic Force Microscopy Imaging

Atomic force microscopy (AFM) imaging was performed in the tapping mode using the Nanoscope IIIa-MultiMode AFM device (Digital Instruments, Santa Barbara, CA, USA) and silicon cantilevers (Bruker OTESPA-R3) with the nominal resonance frequency of 300 kHz and a nominal tip radius of 7 nm. Oligonucleotide solutions for AFM imaging were prepared by diluting the solutions used for DLS measurements with an imaging buffer (10 mM KCl + 10 mM MgCl_2_) to the concentrations of either 5 μM or 20 μM [21]. The diluted solution was drop-cast onto the substrate (freshly cleaved V-1 grade muscovite mica) and left to adsorb for 15 min. After this, the excess material was removed by washing the substrate with distilled water. Then, the sample was left to dry for at least one day at ambient conditions. AFM images were recorded on several different parts of the substrate. The obtained images were reproducible in the large areas. Particle height analysis was performed with the particle analysis option of the NanoScope Analysis software. To obtain the length distribution, the images were analyzed with the ImageJ program [49,50] and the Ridge Detection plugin [51]. The latter is based on the algorithm for detecting ridges and lines as described by Steger [52]. Both analysis methods were applied on a number of images and the cumulative statistics is presented. In the case of d(G_4_C_2_), length statistics were obtained by limiting the detection to elongated objects with a height equal or greater than 1 nm. To identify the artefacts from residual buffer salts in AFM images a set of measurements with only the buffering salt on mica was made. Drop cast films without water rinsing had circular structures of varying diameter and height (many of them much higher than 2 nm). After rinsing with distilled water, nearly all surface structures disappeared. We can therefore assume that circular structures observed in Figure 4 are not made of salt. Nevertheless, a small amount of salt could still be attached to the G-quadruplex structures and affect the AFM measurements.

## 5. Conclusions

Based on our DLS and AFM measurements no generalization of the expected self-assembly of d(G_4_C_2_)*_n_* repeats could be made. We found, however, that all the studied repeats form much larger aggregates than previously reported due to a strong tendency for π-π stacking of the basic quadruplex units. The assembly of numerous d(G_4_C_2_)_n_ repeats, which are found in the defected gene of patients with ALS and FTD, could therefore be governed by the same assembling pattern: first the formation of basic, smaller G-quadruplexes, followed by stacking of these quadruplexes in a “beads on a string” fashion. Thus, DLS together with AFM was able to provide new and important insights into the folding scheme and supramolecular assembly of the highly polymorphic d(G_4_C_2_)*_n_* sequences.

## Data Availability

The data presented in this study are available on request from the corresponding author.
